# Outstanding Reviewers for *RSC Chemical Biology* in 2024

**DOI:** 10.1039/d5cb90026c

**Published:** 2025-07-08

## Abstract

We would like to take this opportunity to thank all of *RSC Chemical Biology*’s reviewers for helping to preserve quality and integrity in chemical science literature. We would also like to highlight the Outstanding Reviewers for *RSC Chemical Biology* in 2024.

## Abstract 2

We would like to take this opportunity to highlight the Outstanding Reviewers for *RSC Chemical Biology* in 2024, as selected by the editorial team for their significant contribution to the journal.

## Graphical abstract

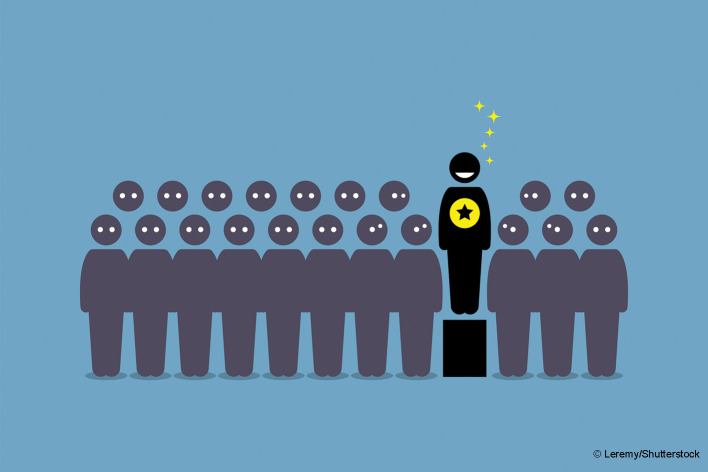

Each one of our outstanding peer reviewers has been carefully selected by our editorial team and includes active researchers who have made significant contributions to peer review and have gone above and beyond in their actions. Our outstanding reviewers from 2024 have been chosen based on several different measures, including the number, timeliness and quality of the reports completed over the last 12 months. In addition to reviewers who provided a high number of quality reports, we are pleased to highlight reviewers who provided exceptionally thorough and detailed reports and reviewers who made additional efforts to aid authors in improving their manuscripts.

Dr Andreas Brunschweiger

Technische Universität Dortmund

ORCID: 0000-0002-4401-1495

Dr Jeroen Dickschat

University of Bonn

ORCID: 0000-0002-0102-0631

Dr Matthew Griffin

University of California Irvine

ORCID: 0000-0001-9549-4418

Dr Tom McAllister

Newcastle University

ORCID: 0000-0001-6906-4510

Professor Michal Hocek

Czech Academy of Sciences

ORCID: 0000-0002-1113-2047

Dr Gerbrand van der Heden van Noort

Leiden University Medical Centre

ORCID: 0000-0001-5955-6431

Dr Elah Pick

University of Haifa

Dr Lianne Willems

University of York

ORCID: 0000-0001-5411-1329

Dr Ru Yan

University of Macau

ORCID: 0000-0001-6268-360X

Professor Oliver Zerbe

University of Zurich

ORCID: 0000-0003-0475-438X

We would also like to thank the *RSC Chemical Biology* Editorial Board and Advisory Board and the chemical biology community for their continued support of the journal, as authors, reviewers and readers.

We continue to work on improving the diversity of our reviewer pool to reflect the diversity of the communities that we serve.

Dr Anna Rulka, Executive Editor

Dr Viktoria Titmus, Editorial Manager

